# Daily management of gliomas, glioneuronal, and neuronal tumors in the era of the 2021 WHO classification of nervous tumors

**DOI:** 10.3389/fneur.2024.1407572

**Published:** 2024-07-29

**Authors:** Mona Mlika, Moncef Mokni, Faouzi Mezni, Soumeya Rammeh

**Affiliations:** ^1^Faculty of Medicine of Tunis, University of Tunis El Manar, Tunis, Tunisia; ^2^Department of Pathology, Trauma and Major Burn Center, Tunis, Tunisia; ^3^Department of Pathology, Farhat Hached Hospital, Sousse, Tunisia; ^4^Department of Pathology, Charles Nicolle Hospital, Tunis, Tunisia

**Keywords:** World Health Organization, nervous tumors, methyloma, molecular diagnosis, diagnosis

## Background

The World Health Organization (WHO) classification of tumors in the central nervous system (CNS) serves as a reference standard for pathologists. Thus, classification is frequently revised to incorporate advances in knowledge. However, during each review, a balance must be maintained between the introduction of new entities and the recognition of certain diagnostic or prognostic biomarkers while ensuring these markers and techniques remain globally accessible and maintain relative continuity.

We will explore the historical context of the different revisions of the WHO classifications of CNS tumors, with particular attention to the 2016 classification and the contributions of the c-IMPACT-NOW (Consortium to Inform Molecular and Practical Approaches to CNS Tumor Taxonomy) (1, 2). We will then focus on the 2021 classification, highlighting the main conceptual changes, the role of advanced technologies, especially methylome, and the specific changes, including the description of new entities (referred to as “types” in the 2021 classification) (2, 3).

## Set of classifications

The first WHO classification of CNS tumors was published in 1979, followed by the second edition in 1993. The third edition, published in 2000, was titled Pathology and Genetics and was edited by pathologist P. Kleihues and geneticist W. Cavenee. Significant advances in understanding genetic alterations, notably the identification of key genes in gliomagenesis, marked the fourth edition in 2007. Key genes included the isocitrate dehydrogenase (IDH) 1/2 genes for adult diffuse gliomas (4) and the histone genes H3F3A and HIST1H3B/C for median diffuse gliomas, occurring mainly in children (5).

At the same time, the field of embryonic tumors was also full of key publications with the identification of recurrent alterations in medulloblastomas, allowing classification into three groups based on the activation of two major molecular pathways: WNT and Sonic Hedgehog (SHH). These advancements necessitated a revision of the fourth WHO classification of CNS tumors. However, to align with the blue books of other organs, a fifth edition was not proposed. Instead, a revised version of the fourth edition was published in 2016 (6).

Finally, in 2021, the fifth edition was published. The main changes in the 2016 classification focused on diffuse astrocytic and oligodendroglial gliomas. Within this group, adult diffuse gliomas could benefit from a histomolecular diagnosis. Three types of gliomas were defined: IDH mutant gliomas, further divided into astrocytomas IDH mutant (whose diagnosis was based on the absence of 1p/19q co-deletion and the presence of an IDH mutation) and oligodendrogliomas IDH mutant and 1p/19q co-deleted, and IDH-wildtype gliomas, primarily represented by glioblastomas, IDH-wildtype.

In addition, the term “NOS” (not otherwise specified) was introduced for cases where a precise molecular diagnosis could not be made. This can be due to multiple reasons, such as the inability to perform necessary molecular techniques, technical failure, or inconsistent molecular results. The entity “mixed oligoastrocytic glioma” was deleted and could only be classified as “NOS.”

Within the category of diffuse astrocytomas and oligodendroglial gliomas, a new histomolecular entity was introduced: diffuse midline glioma with the H3 K27M mutation. Another significantly revised category was embryonic tumors, which use a dual approach to classifying medulloblastoma based on histopathology and genetic data.

### The successive contributions of cIMPACT-NOW updates

The cIMPACT-NOW consortium, comprising a steering committee of 12 pathologists and 6 international clinicians, was formed at the initiative of D.N. Louis and A. Von Deimling immediately after the publication of the 2016 WHO classification (7). Its purpose was to publish regular updates to improve the classification of CNS tumors, considering the rapid advances in knowledge. Indeed, limitations in the 2016 WHO classification quickly became apparent, and waiting for the publication of a new edition (with an unknown date) seemed detrimental to the management of patients with CNS tumors. It is important to note that this consortium has clearly indicated that it does not replace the WHO. Thus, the term NOW has two meanings: literally “now” and also “Not Official WHO.”

Since its creation, seven updates have been published by c-IMPACT-NOW dealing with specific themes. The themes were chosen by the members of the steering committee, and additional pathologists (or clinicians) were invited to join this committee to participate in the discussion on specific topics.

The main themes were:

• Clarification of terminology (8, 9);

• The grading of gliomas and, particularly, considering certain molecular criteria for classifying a grade II or III IDH-wildtype glioma as a grade IV molecular “glioblastoma” (amplification of EGFR and/or mutation of the TERT promoter and/or presence of a gain of chromosome 7 and a loss of chromosome 10) (10);

• Consideration of homozygous deletion of CDKN2A in the grading of IDH mutant gliomas (11);

• Recognition and histomolecular classification of diffuse low-grade gliomas with altered *MYB, MYBL1, or FGFR1* genes, or BRAF mutation, V600E (12);

• Histomolecular classification of ependymomas (13);

• A summary of the conclusions from the Utrecht meeting of September 2019, proposing the inclusion of new entities and guidelines for the future WHO classification of CNS tumors (14).

### 2021 classification: major conceptual changes

An effort to simplify and harmonize with the WHO classification of other organ diseases was made:

• The term “entity” is replaced by “type” and “variant” by “subtype;” whenever possible, the title of a tumor type has been shortened. For example, the tumor labeled “third ventricular choroid glioma” became “choroid glioma,” and the location of this tumor type was given in the definition. In addition, to standardize, the definition of each tumor type has two components: one specifying the histopathological characteristics and another reporting the characteristic molecular alteration(s).

• The grade is given in Arabic numerals and must be associated with the WHO grade of CNS tumors (“WHO CNS grade”).

• The number of mitoses should be given per mm^2^ and not per 10 high power fields (HPF) because of disparities in HPF sizes. This correspondence was difficult to establish retrospectively for all CNS tumors, as some older publications did not detail this type of information.

Contrary to previous WHO classifications, where each entity was associated with a grade, in the 2021 WHO classification, the same tumor type can be associated with different grades. For example, astrocytoma, IDH mutant could be graded as grade 2, 3, or 4, and oligodendroglioma IDH mutant and 1p/19q co-deleted could be graded as grade 2 or 3. Finally, grading criteria can be mixed: histopathological and molecular. For example, homozygous deletion of CDKN2A is included in the grading of IDH mutant astrocytomas, with its presence conferring a grade 4, regardless of the histopathological aspect of the tumor.

Thus, the symbols of the genes are italicized to differentiate them from the proteins and gene families, the latter being in Roman font (straight characters). The sequence variation nomenclature follows the recommendations of the Human Genome Variation Society (HGVS) (https://varnomen.hgvs.org/). The ratio of each variation is established with respect to a reference sequence corresponding to the coding sequence of the gene, preceded by the prefix “c.” and with respect to the corresponding predicted sequence, preceded by the prefix “p.” No specific recommendations are made concerning the technique to be used to search for a genetic alteration of interest.

In the last decade, new molecular techniques, whether targeted [immunohistochemistry with antibodies recognizing a mutated protein; fluorescence *in situ* hybridization (FISH); digital PCR targeting mutations; or restricted NGS panel DNA or RNA (fusion genes)] or non-targeted (RNAseq, whole exome sequencing, whole genome sequencing), have shown their utility in searching for molecular alterations associated with a given tumor type. The methyloma analysis technique has been developed as a powerful tool for classifying CNS tumors. Indeed, the methylation largely depends on the cell of origin, its location, and any associated molecular alterations. It is relatively stable between the initial tumor and recurrences.

The DKFZ (Deutsches Krebsforschunszentrum: German Cancer Research Center) team in Heidelberg has built a classification using artificial intelligence to classify CNS tumors according to their methylation profile. A number of methylation (MC) classes were initially proposed from a series of more than 2000 tumors classified according to 2016 WHO classification. This tool was made freely available (https://www.molecularneuropathology.org/mnp) for download. When the prediction score is >0.9, the diagnosis proposed by the methylation class is generally reliable. A score < 0.3 is given when no methylation class can be proposed. Finally, when the score is between 0.3 and 0.9, the methionization class can be informative if it is compatible with the morphology, the immunohistochemical, and the molecular profile.

Major changes are noted in the 2021 WHO classification with the introduction of 22 new tumor types, mainly involving tumors in children and young adults, and the creation of a new large tumor category titled “gliomas, glioneuronal, and neuronal tumors.” The 2021 WHO classification of gliomas, glioneuronal, and neuronal tumors is represented in Table 1.

**Table 1:** The classification of gliomas, glioneuronal tumors, and neuronal tumors according to the fifth edition of the World Health Organization classification.

**Adult-type diffuse gliomas**	- Astrocytoma, IDH mutant - Oligodendroglioma, IDH mutant, and 1p/19q codeleted - Glioblastoma, IDH-wildtype
**Pediatric-type diffuse low-grade gliomas**	- Diffuse astrocytoma, MYB or MYB-L1 altered - Angiocentric glioma - Polymorphous low-grade neuroepithelial tumor of the young - Diffuse low-grade glioma; MAPK pathway altered
**Pediatric-type diffuse high-grade gliomas**	- Diffuse midline glioma, H3K27-altered - Diffuse hemispheric glioma, H3G34-mutant - Diffuse pediatric-type high-grade glioma, H3-wildtype, and IDH-wildtype - Infant-type hemispheric glioma
**Circumscribed astrocytic gliomas**	- Pilocytic astrocytoma - High-grade astrocytoma with piloid features - Pleomorphic xanthoastrocytoma - Subependymal giant-cell astrocytoma - Chordoid glioma - Astroblastoma MN1-altered
**Glioneuronal and neuronal tumors**	- Ganglioglioma - Gangliocytoma - Desmoplastic infantile ganglioglioma/astrocytoma - Dysembryoplastic neuroepithelial tumor - Diffuse glioneuronal tumor with oligodendroglioma-like features and nuclear clusters - Papillary glioneuronal tumor - Rosette-forming glioneuronal tumor - Myxoid glioneuronal tumor - Diffuse leptomeningeal glioneuronal tumor - Multinodular and vacuolating neuronal tumor - Dysplastic cerebellar gangliocytoma - Central neurocytoma - Extraventricular neurocytoma - Cerebellar liponeurocytoma
**Ependymal tumors**	- Supratentorial ependydoma - Supratentorial ependymoma, ZFTA-fusion positive - Supratentorial ependymoma, YAP1-fusion positive - Posterior fossa ependymoma - Posterior fossa group A (PFA) ependymoma - Posterior fossa group B (PFB) ependymoma - Spinal ependymoma - Spinal ependymoma, MYCN-amplified - Myxopapillary ependymoma - Subependymoma

#### Gliomas, glioneuronal tumors, and neuronal tumors

The 2021 WHO classification includes under the category “gliomas, glioneuronal, and neuronal tumors” several tumor groups formerly called gliomas, diffuse astrocytomas and oligodendrogliomas, other astrocytic tumors, other gliomas, neuronal and glioneuronal tumors, and ependymomas. Within this new large category, gliomas are classified based on their circumscribed or diffuse nature, typical age of occurrence, and aggressiveness. Three main groups of diffuse gliomas have been defined: one for adult gliomas (which can be grade 2, 3, or 4) and two pediatric subtypes (either low-grade or high-grade).

Adult diffuse gliomas include three tumor types: IDH mutant diffuse astrocytomas (grade 2, 3, or 4), IDH mutant and 1p/19q co-deleted oligodendrogliomas (grade 2 or 3), and glioblastomas, IDH-wildtype (grade 4). The term “glioblastoma” is no longer used for IDH mutant gliomas and is replaced by the term “grade 4 IDH mutant astrocytoma.” Each type of diffuse glioma is characterized by recurrent molecular alterations, some of which are essential for diagnosis, while others are not.

Moreover, for IDH mutant astrocytomas, grading is based on both histopathological and molecular features. For example, the presence of a homozygous deletion of CDKN2A necessitates classifying the glioma as grade 4, regardless of histopathological data. The diagnosis of glioblastoma is based either on the absence of IDH mutation in a diffuse glioma with endothelial-capillary proliferation and/or necrosis, or in the absence of these histopathological criteria, by the identification of at least one of the following three alterations: mutation of the TERT promotor, the combination of a gain of chromosome 7 and a loss of chromosome 10 (+7/10), or amplification of EGFR (3).

Unfortunately, the WHO (3) classification does not suggest a mitosis threshold to distinguish grade 2 from grade 3 IDH mutant astrocytomas due to the absence of robust publications on this subject. Similarly, there is no precise mitosis threshold for diagnosing anaplastic oligodendroglioma IDH mutant 1p/19q co-deleted in the absence of endothelial-capillary proliferation. However, a minimum threshold of six mitoses, as recommended by some authors, is cited. The presence of a homozygous deletion of CDKN2A in an IDH-mutated and 1p/19q co-deleted oligodendroglioma was not considered for grading these tumors, despite the very poor prognosis associated with this alteration in the French POLA cohort POLA (14).

The high-grade pediatric diffuse gliomas include four tumor types, three of which are new, where age and location are important criteria: diffuse midline glioma with H3 K27 alteration, diffuse hemispherical glioma H3 G34-mutated, high-grade pediatric diffuse glioma H3 and IDH wildtype, and, finally, the infantile hemispherical glioma.

The diffuse midline glioma with H3 K27 alteration is characterized by a loss of nuclear expression of H3K27 [the trimethylated form on lysine (K) at position 27 of the protein H3] (15). This loss of expression is associated with either an H3 K27 mutation, an EGFR mutation (frequent in bi-thalamic localization), or an overexpression of EZHIP (16). These gliomas are classified as grade 4, regardless of histopathological aspects.

The diffuse hemispherical glioma H3 G34-mutant does not express OLIG2 and shows a loss of ATRX nuclear expression and p53 overexpression (17). Infantile hemispheric glioma is associated with fusions involving the NTRK1/2/3, ALK, ROS1, or MET (18) genes. Their clinical presentation is often similar to that of childhood desmoplastic gangliogliomas, which represent the main differential diagnosis.

Diffuse low-grade pediatric gliomas include four tumor types, three of which are new entities: diffuse astrocytoma with alterations in MYB or MYBL1, angiocentric glioma (most often associated with MYB-QKI fusion), polymorphous low-grade neuroepithelial tumor of the young (PLNTY) (19), and low-grade diffuse gliomas with alterations in the MAP kinase pathway (mainly FGFR or BRAF) (12). This group presents clinical (epileptogenicity, young age of occurrence), phenotypic (CD34 positivity), radiological (cortical localization), and molecular (alteration of the MAP Kinases pathway) similarities with certain glioneuronal tumors (20).

The circumscribed astrocytic glioma group includes pilocytic astrocytoma, pleomorphic xanthoastrocytoma, subependymal giant cell astrocytoma, chordoid glioma, and two new tumor types: anaplastic astrocytoma with piloid aspects (HGAP for high-grade astrocytoma with piloid features), whose diagnosis is based on methyloma (21), and astroblastoma with MN1 alteration (22).

Glioneuronal and neuronal tumors contain 14 different types: ganglioglioma, gangliocytoma, infantile desmoplastic ganglioglioma/infantile desmoplastic astrocytoma, dysembryoplasic neuroepithelial tumor, papillary glioneuroal tumor, glioneuronal tumor with rosettes, dysplastic gangliocytoma of the cerebellum (Lhermitte Duclos disease), central neurocytoma, extraventricular neurocytoma, and liponeurocytoma of the cerebellum. In addition, two new tumor types are described: the myxoid glioneuronal tumor (localized at the septum pellucidum, whose morphology mimics a dysembryoplastic neuroepithelial tumor but with a mutation of the PDFGRA gene) (23), and the multinodular and vacuolated neuronal tumor (associated with a recurrent alteration of the MAPKinase pathway, most commonly MAP2K1) (24, 25). A third type is classified as provisional and noted in italics: “glioneuronal tumor with oligo-like aspects and clusters of nuclei” (DGONC for diffuse glioneuronal tumor with oligodendroglioma-like features and nuclear clusters), whose diagnosis is based on methyloma and frequent monosomy of chromosome 14 (26).

This broad category also includes ependymal tumors that include ependymomas, subependymomas, and myxopapillary ependymomas. Ependymomas are now classified according to their location: supratentorials, infratentorials, and spinal, and within the same location according to the underlying molecular alteration [supratentorials either with ZFTA fusion or YAP1 fusion, and infratentorials either PFA type (posterior fossa A) or PFB type]. The terminology proposed in 2016 for supratentorial ependymomas with C11orf95-RELA fusion has been modified. Alternative pathogenic fusions between C11orf95 (now called ZFTA) and partners other than RELA have been identified, prompting this change.

#### Choroid plexus tumors

There is little change in choroid plexus tumors, but to emphasize their rather epithelial nature, these tumors stand apart as opposed to ependymomas, which are now part of the broad category of gliomas, glioneuronal, and neuronal tumors.

#### Embryonal tumors

In the chapter on embryonic tumors, the location of the lesion is important, especially the posterior fossa vs. other locations. This category includes medulloblastomas and other embryonic tumors. Concerning medulloblastomas, the main molecular types already present in the 2016 WHO classification are retained: medulloblastomas with the activation of the WNT pathway, medulloblastomas with the activation of the SHH pathway (which can be either TP53-mutated or TP53-wildtype), and non-WNT/non-SHH medulloblastomas. However, while the 2021 WHO classification retains these broad categories, it now recognizes different molecular subgroups within each category based on distinct transcriptomic and/or methylation profiles. There are now four subgroups of SHH medulloblastomas (SHH-1 to SHH-4) and eight subgroups of non-WNT/non-SHH medulloblastomas (subgroups 1–8) (19, 27–29). Some of these subgroups are characterized by specific clinicopathological presentations and are of diagnostic, prognostic, or predictive interest for treatment responses. For example, subtypes SHH-1 and SHH-2 are prevalent in young children and often present with nodular extensive histopathology, while certain subtypes of non-WNT/non-SHH medulloblastomas are associated with therapeutic resistance (30, 31).

Furthermore, the 2021 WHO classification no longer distinguishes the previously defined histological types: classical, nodular/desmoplasic, extensive nodular, and large cell/anaplastic. These histological subtypes are now considered specific histopathological presentations listed in the chapter *medulloblastoma defined based on microscopic features*. Histomolecular diagnosis “in strata” is particularly appropriate for the group of medulloblastomas. Regarding the group of other embryonic tumors, two new histomolecular types are identified following the work of Sturm et al. (22), for the diagnosis of embryonic tumors.

#### Tumors of the pineal parenchyma

A new tumor type has been recognized in addition to the four known tumor types [pineal parenchymal tumor of intermediate differentiation (PPTID), pineoblastoma, and papillary tumor of the pineal region]: the “desmoplasic myxoid tumor of the pineal region with SMARCB1 mutation” (32). Moreover, the presence of an alteration in the KBTBD4 gene is a diagnostic marker for PPTID. Finally, in pinealoblastomas, several molecular subtypes, whose diagnosis is based on methyloma, are identified (miRNA processing-altered, RB1-altered, and MYC/FOXR2-activated); they are characterized by a different prognosis and age of occurrence.

#### Tumors of the cranial and paraspinal nerves

The main changes observed in this tumoral group include the inclusion of paraganglioma (which is no longer part of glioneuronal and neuronal tumors) and the recognition of paraganglioma of the ponytail as a distinct tumor type from paragangliomas observed in other locations, which do not express GATA3 (33). By analogy to the classification of soft tissue tumors, the aggressive nature of melanotic schwannoma is recognized, and this tumor is now called a malignant melanocytic nerve sheath tumor. Finally, a new tumor subtype has been listed among neurofibromas: the “atypical neurofibromatous tumor of unknown biological potential” (ANNUBP), which is associated with neurofibromatosis type 1 and has a characteristic methylation profile (34).

#### Meningiomas

Meningiomas now represent a single tumor type with 13 histopathological subtypes, classified as grade 1, 2, or 3. Grading criteria are applied regardless of the subtype.

#### Mesenchymal tumors non-meningothelial

Three new tumor types are now recognized: “intracranial mesenchymal tumor with FET-CREB fusion” (a provisional type, corresponding to tumors previously called either angiomatoid fibrous histiocytoma or intracranial myxoid mesenchymal tumor) (35, 36), “sarcoma with CIC rearrangement” (22), and “primary intracranial sarcoma with DICER1 mutation” (37). The term hemangioma pericytoma has been definitively replaced by “solitary fibrous tumor” (grade 1, 2, or 3).

#### Lymphomas and histiocytic tumors

Few modifications involved this group of tumors. The lymphoma group contains two subgroups: CNS lymphomas and miscellaneous rare lymphomas. The former consists of primary diffuse large-B-cell lymphoma, immunodeficiency-associated CNS lymphomas, lymphomatoid granulomatosis, and intravascular large-B-cell lymphoma. The latter includes MALT lymphoma of the dura, other low-grade B-cell lymphomas of the CNS, anaplastic large-cell lymphoma, and T-cell and NK/T-cell lymphomas. Histiocytic tumors are composed of Erdheim-Chester disease, Rosai-Dorfman disease, juvenile xanthogranuloma, Langerhans cell histiocytosis, and histiocytic sarcoma.

#### Tumors of the sellar region

Two histopathological types are now retained for craniopharyngiomas (and not two morphological subtypes, as in 2016), justified by distinct clinical, histopathological, and genetic characteristics: “adamantinomatous craniopharyngioma” (associated with a clonal mutation of CTNNB1) (38) and “papillary craniopharyngioma” (with a BRAF mutation) (39). Conversely, because of their histopathological relationship and common expression of TTF1, only one tumor type is retained under the label “pituicytoma/granular cell tumor/fusiform cell oncocytoma” (40, 41). A new chapter is now dedicated to pituitary adenomas, called “neuroendocrine pituitary tumors (PitNET),” following the WHO classification of endocrine tumors. Finally, a new tumor type has been introduced: the “blastoma of the hypophysis,” associated with DICER1 mutations.

## Discussion

**Figure 1:**
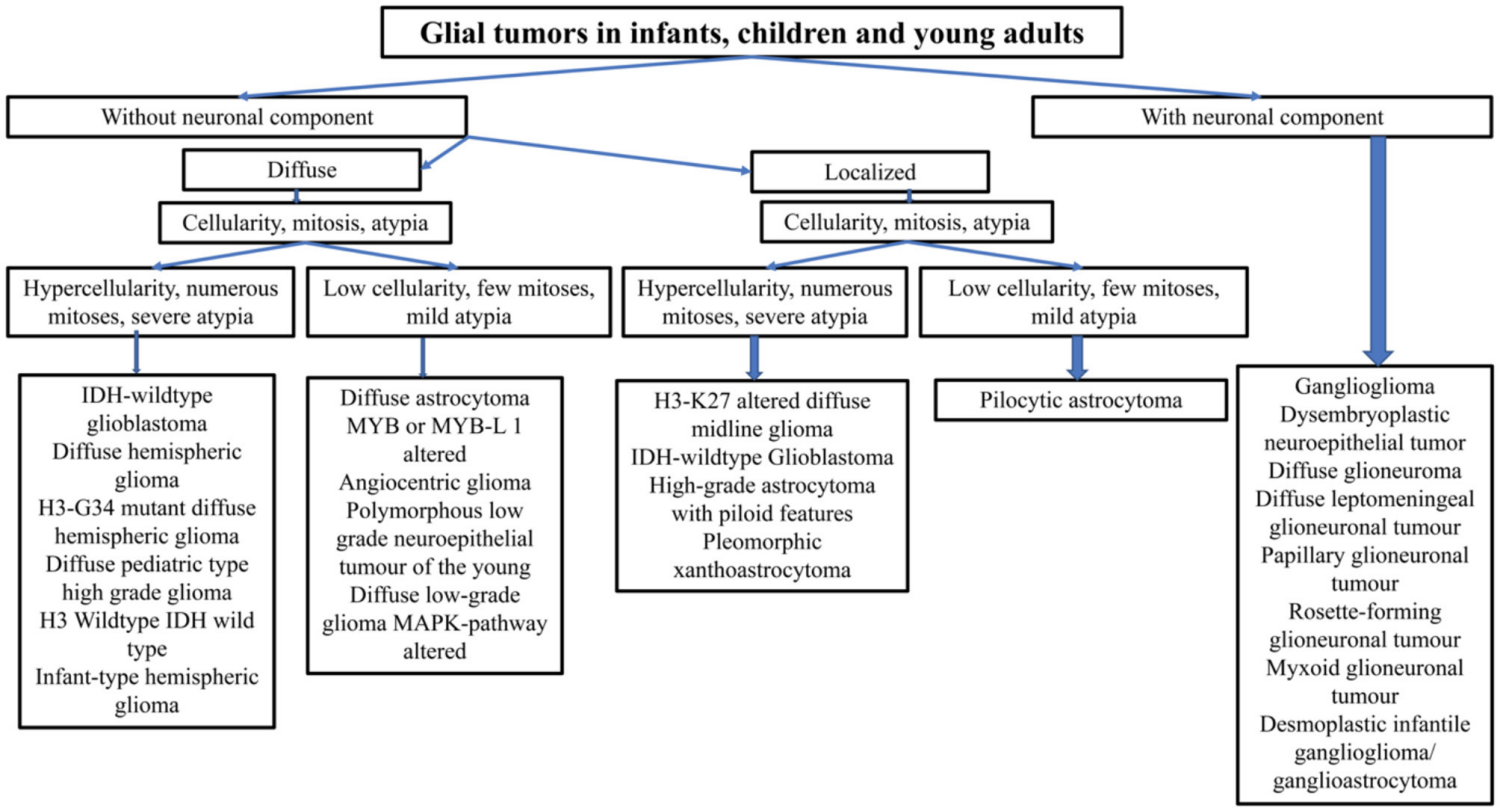
Practical decisional algorithm based on the clinical and morphological features of gliomas in children and young adults.

**Figure 2:**
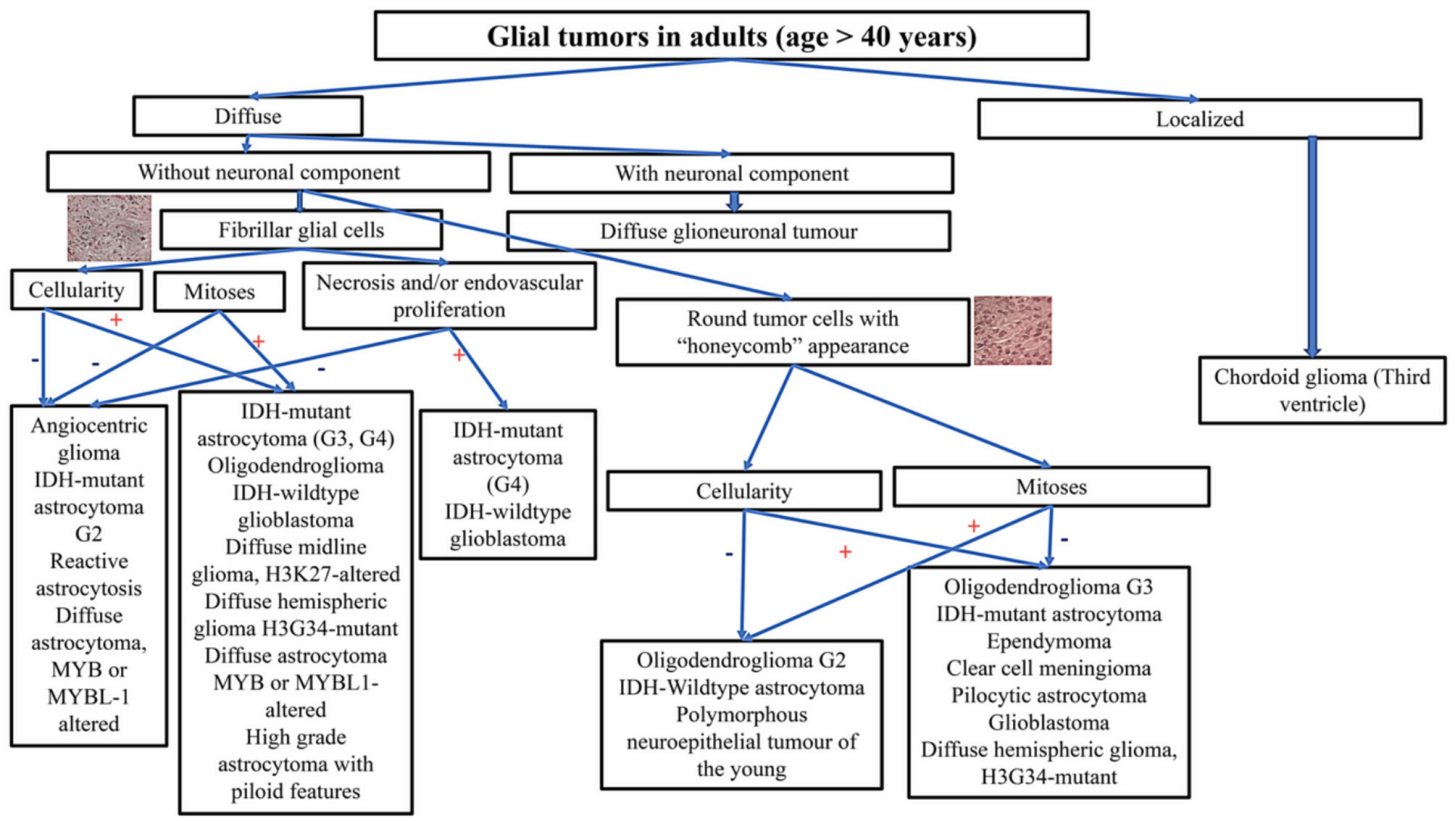
Practical decisional algorithm, based on the clinical and morphological features of gliomas, for adults aged more than 40 years.

**Figure 3:**
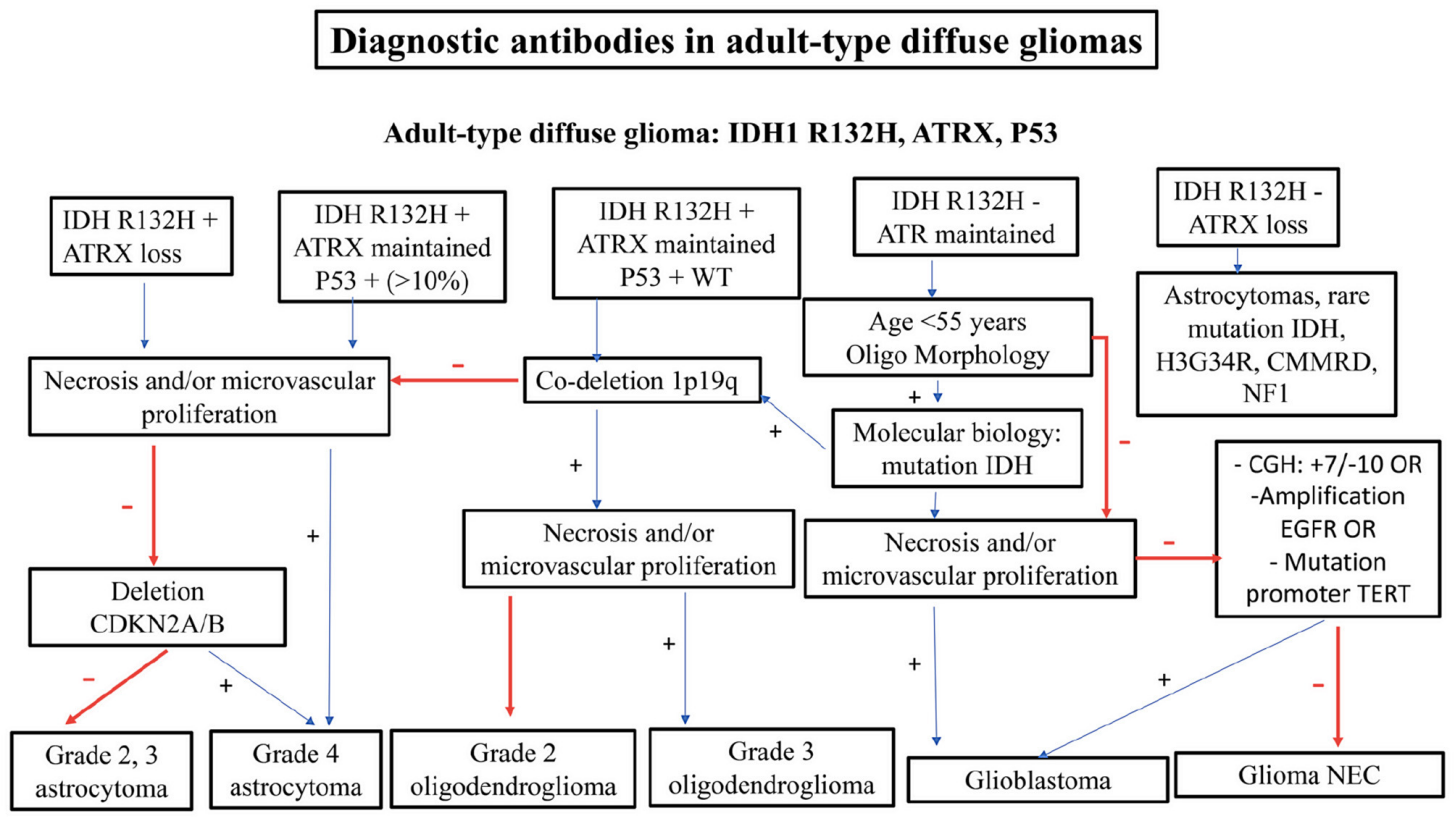
Practical approach for immunohistochemical studies in order to differentiate between the different adult-type diffuse gliomas.

When analyzing a glial tumor, after considering its localization and the age of the patient, the first step involves screening the cellularity, morphology, architecture, proliferation index, vascularization, and necrosis. However, this first step may be challenging, especially in small biopsies. Immunohistochemistry is useful for differentiating gliomas due to its low cost and minimal material requirements. The limitations of immunohistochemistry include the limited number of antibodies available and its high sensitivity to ischemia, necrosis, and electrocoagulation, which increase the risk of false negative cases and diagnostic errors. Practical decisional algorithms based on the clinical and morphological features of gliomas for children, young adults, and adults over 40 years are represented in Figures 1, 2 (3, 42). Figure 3 illustrates a practical approach for immunohistochemical studies to differentiate between the different adult-type diffuse gliomas (43, 44).

A panel of antibodies, including IDH1R132H, ATRX, and P53, may be useful in routine practice for differentiating between grade 2, 3, and 4 astrocytomas, grade 2, 3 oligodendrogliomas, glioblastomas, and NEC gliomas. NEC gliomas are IDH1 non-mutant gliomas that express ATRX, lack necrosis or microvascular proliferation, and do not have chromosome 7 gain, chromosome 10 loss, EGFR amplification, or TERT promoter mutation. Glioblastoma is a glial tumor that is IDH1 non-mutant with necrosis and/or microvascular proliferation or with chromosome 7 gain and chromosome 10 loss or EGFR amplification or TERT promoter mutation. Oligodendrogliomas are glial tumors that are IDH1 R132H mutant, express ATRX, and have P53 WT with 1p19q codeletion. Even glial tumors that are IDH1 R132H non-mutant but express ATRX, with IDH mutation assessed using molecular testing and 1p19q codeletion, are considered oligodendrogliomas. Glial tumors that are IDH1 R132H mutant, with or without ATRX expression and with or without P53 expression (>10%), are considered astrocytomas.

Molecular diagnosis plays a key role in the 2021 WHO classification of nervous tumors, especially in pediatric tumors and new entities.

Pediatric tumors are challenging because of the multiplicity of entities, variable prognoses, oncogenetic implications, and mandatory molecular criteria that are often not classical. Cellular density, mitotic activity, and nuclear atypia are less reliable criteria in pediatric tumors. Next-generation sequencing (NGS) techniques are useful for many new entities, with or without methylome analysis. Table 2 illustrates the molecular techniques that should be used in the different molecular new entities.

**Table 2:** The molecular techniques that should be used in the different molecular new entities.

**Gliomas, glioneuronal tumors, and neuronal tumors**		**Molecular techniques**
**Adult-type diffuse gliomas**	- Astrocytoma, IDH mutant - Oligodendroglioma, IDH mutant and 1p/19q codeleted - Glioblastoma, IDH-wildtype	- NGS ARCHER - NGS ARCHER + FISH ou SNParray - NGS ARCHER
**Pediatric-type diffuse low-grade gliomas**	- Diffuse astrocytoma, MYB, or MYB-L1 altered - Angiocentric glioma - Polymorphous low-grade neuroepithelial tumor of the young - Diffuse low-grade glioma; MAPK pathway altered	- FISH ou NGS panel DRAGON - NGS panel DRAGON - NGS ARCHER + METHYLOMA - NGS ARCHER
**Pediatric-type diffuse high-grade gliomas**	- Diffuse midline glioma; H3K27-altered - Diffuse hemispheric glioma, H3G34-mutant - Diffuse pediatric-type high-grade glioma, H3-wildtype, and IDH-wildtype - Infant-type hemispheric glioma	- NGS panel DRAGON - Targeted techniques - NGS panel DRAGON + METHYLOMA - NGS ARCHER + METHYLOMA
**Circumscribed astrocytic gliomas**	- Pilocytic astrocytoma - High-grade astrocytoma with piloid features - Pleomorphic xanthoastrocytoma - Subependymal giant-cell astrocytoma - Chordoid glioma - Astroblastoma MN1-altered	- NGS ARCHER - METHYLOMA - NGS ARCHER + FISH +/METHYLOMA - - NGS panel DRAGON - FISH + METHYLOMA
**Glioneuronal and neuronal tumors**	- Ganglioglioma - Gangliocytoma - Desmoplastic infantile ganglioglioma/astrocytoma - Dysembryoplastic neuroepithelial tumor - Diffuse glioneuronal tumor with oligodendroglioma-like features and nuclear clusters - Papillary glioneuronal tumor - Rosette-forming glioneuronal tumor - Myxoid glioneuronal tumor - Diffuse leptomeningeal glioneuronal tumor - Multinodular and vacuolating neuronal tumor - Dysplastic cerebellar gangliocytoma - Central neurocytoma - Extraventricular neurocytoma - Cerebellar liponeurocytoma	- NGS ARCHER - - NGS ARCHER + METHYLOMA - ddPCR - Ch14 monosomy - NGS DRAGON - NGS DRAGON - NGS DRAGON - NGS ARCHER + METHYLOMA - NGS ARCHER - NGS DRAGON - - NGS ARCHER + METHYLOMA -

While NGS has many advantages, it remains very expensive, especially in low-income countries. Encouraging the implementation of more affordable techniques, including immunohistochemistry, could be a solution in routine practice.

Accurate diagnosis of nervous tumors is mandatory due to their prognostic implications. Additionally, more specific therapies can be designed when histologically similar but molecularly distinct tumors are accurately diagnosed.

## Conclusion

The 2021 WHO classification of nervous tumors introduced many modifications, particularly concerning glial tumors in both adults and children. Implementing molecular features in daily practice is challenging, especially in low-income countries. More immunohistochemical markers need to be assessed as surrogates for molecular techniques.

## Author contributions

MMl: Conceptualization, Software, Supervision, Validation, Visualization, Writing – original draft, Writing – review & editing. MMo: Conceptualization, Writing – original draft, Writing – review & editing. FM: Formal analysis, Investigation, Writing – original draft, Writing – review & editing. SR: Conceptualization, Supervision, Writing – original draft, Writing – review & editing.
